# From first generation biofuels to advanced solar biofuels

**DOI:** 10.1007/s13280-015-0730-0

**Published:** 2015-12-14

**Authors:** Eva-Mari Aro

**Affiliations:** Department of Biochemistry, Molecular Plant Biology, University of Turku, 20014 Turku, Finland

**Keywords:** Advanced biofuel, Biomass, Photosynthesis, Photosynthetic microorganism, Solarfuel, Synthetic biology

## Abstract

Roadmaps towards sustainable bioeconomy, including the production of biofuels, in many EU countries mostly rely on biomass use. However, although biomass is renewable, the efficiency of biomass production is too low to be able to fully replace the fossil fuels. The use of land for fuel production also introduces ethical problems in increasing the food price. Harvesting solar energy by the photosynthetic machinery of plants and autotrophic microorganisms is the basis for all biomass production. This paper describes current challenges and possibilities to sustainably increase the biomass production and highlights future technologies to further enhance biofuel production directly from sunlight. The biggest scientific breakthroughs are expected to rely on a new technology called “synthetic biology”, which makes engineering of biological systems possible. It will enable direct conversion of solar energy to a fuel from inexhaustible raw materials: sun light, water and CO_2_. In the future, such solar biofuels are expected to be produced in engineered photosynthetic microorganisms or in completely synthetic living factories.

## Introduction

Biofuels are derived from biological material, presently mainly from plants, microorganisms, animals and wastes. All biofuels have the same basic and renewable origin. They are based by the “present-day” photosynthetic conversion of solar energy to chemical energy, which sets them apart from fossil fuels that are based on ancient photosynthesis. In fact, the line between renewable biofuels and non-renewable fossil fuels is sometimes vague and only complete life-cycle analyses in the future will reveal which feedstocks are truly renewable to be used in biofuel production.

Depending on the origin and production technology of biofuels, they are generally called as the first, second and third generation biofuels (according to the EASAC report 2012), while the fourth generation biofuels (as assigned in the present paper) make use of novel synthetic biology tools and are just emerging at the basic research level. On the way towards sustainable bioeconomy, the development of efficient biofuel production strategies based on solar energy is of pivotal importance. This concerns EU in particular, due to limited bioresources. Despite huge advances in photovoltaic technologies for solar energy conversion to electricity, the biofuels are still of primary importance in modern societies, particularly in the transport sector. In this regard, biofuels have become the largest renewable fuel produced and consumed in the world due to increasing demands to replace the fossil fuels with renewable ones, thus reducing greenhouse gas emissions and alleviating climate change. On the other hand, there are serious concerns about competition of land for food and fuel production, particularly due to the fact that the current technology for biofuel production depends on feedstock derived from the edible fraction of food plants (corn, rapeseed, sugar beet and others). Scientific breakthroughs in biofuel production are of key importance in paving the way towards carbon–neutral economy and sustainable biofuel production, including both the quantity and quality of biofuels for different transport sectors.

Today, most of the first generation biofuels are sourced from crop plants as energy-containing molecules like sugars, oils and cellulose. They provide only limited biofuel yields and have a negative impact on food security. Efforts are now needed to accelerate the generation of advanced biofuels by identifying and engineering effective non-food feedstocks, improving the performance of conversion technologies and the quality of biofuels for different transport sectors as well as bringing down the costs (EASAC 2012).

The second generation biofuels are already an improvement in producing biofuels from feedstock of lignocellulosic, non-food materials that include straw, bagasse, forest residues and purpose grown energy crops on marginal lands. Projects are needed to maximise the amount of renewable carbon and hydrogen that can be converted to fuels from “second generation” biomass.

The third generation biofuels are based on algal biomass production. They are presently under extensive research in order to improve both the metabolic production of fuels and the separation processes in bio-oil production to remove non-fuel components and to further lower the production costs.

The fourth generation biofuels—photobiological solar fuels and electrofuels—are expected to bring fundamental breakthroughs in the field of biofuels. Technology for production of such solar biofuels is an emerging field and based on direct conversion of solar energy into fuel using raw materials that are inexhaustible, cheap and widely available. This is expected to occur via revolutionary development of synthetic biology as an enabling technology for such a change. The synthetic biology field is still in its infancy and only a few truly synthetic examples have been published thus far (see Cameron et al. 2014 for a review). For successful progress, one needs to discover new-to-nature solutions and construct synthetic living factories and designer microorganisms for efficient and direct conversion of solar energy to fuel. Likewise, a combination of photovoltaics or inorganic water-splitting catalysts with metabolically engineered microbial fuel production pathways (electrobiofuels) is a powerful emerging technology for efficient production and storage of liquid fuels.

## Current situation

First generation biofuels—direct food-crop-based biofuels—are likely to be largely banned in EU. In its rigorous study, EASAC finds that ‘first generation’ biofuels, once all impacts of biomass cultivation are taken into account, appear to provide little or none of the greenhouse gas reductions required in the Directive whilst putting food, agriculture and natural ecosystems at risk (EASAC 2012). Although the Brazilian sugarcane ethanol is regarded by some specialists as the only biofuel currently produced at commercial scale to meet advanced non-cellulosic renewable fuel targets, there is a lot of criticism concerning the environmental impact, among others. The EASAC report (2012) urges systematic research into the anticipated improvements from second generation biofuels sourced from inedible parts of plants and recommends further development of third generation biofuels extracted from algae as an appropriate means to enhance biofuel production. However, far more innovative solutions—the fourth generation biofuels or direct solar biofuels and synthetic biology technologies—are pertinently needed for replacement of all fossil fuels. Bioengineering of photosynthetic microorganisms towards direct solar fuel production, i.e. the fuel production without a biomass phase, has a history of over 10 years. However, only recently the tools of synthetic biology for efficient and thorough engineering of novel metabolic pathways for fuel and chemical production, as well as optimising the host, have become available also for photosynthetic microorganisms.

## Expected scientific breakthroughs in production of photosynthesis-based biofuels

### Improvement of photon-to-fuel conversion efficiency (PFCE)

Term PFCE is used here to describe the percentage of the energy of photons hitting the organism, converted to chemical energy by photosynthetic light reactions and subsequently, via a number of processes, ending up in the fuel. This terminology was chosen to make some rough comparisons between the efficiency of production of electricity by solar cells (Inganäs and Sundström 2016) and the production efficiency of biofuels by a number of different production systems.

The photon-to-fuel conversion efficiency—including the costs of biomass processing—by the most “sustainable” current feedstocks stands only at 0.16 % for Brazil sugarcane ethanol and ca. 0.15 % for biodiesel from palm oil (see Gouveia 2011) These percentages are much higher than those for transport biofuels from European wheat and rapeseed. Current research aims at higher PFCEs and better quality as absolute requirements for sustainable biofuel production. Developments of various second and third generation biofuels, for example, with improvements in biomass processing technologies and by search for better feedstocks, like algal biomass with inherently higher photon-to-biomass conversion efficiency than in land plants, are important steps in this direction. Fourth generation biofuels are likely to bring much higher improvements in the PFCE. For example, the future photobiological solar fuel production system aims at using photosynthetic microorganism only as a “catalyst”: it harvests the solar energy and uses this to direct production of a high-quality fuel in high yield. In optimal cases, the microorganism will also be tailored to secrete the fuel, making it possible to collect the fuel continuously in a photo-bioreactor. In the future direct solar fuel production technology, the fuel production is not based on harvested biomass; on the contrary, in an ideal system the biomass production is halted when the system is shifted to direct photobiological solar fuel production. Thus, the growth of microorganisms is aimed to be temporarily separated from the fuel production process. To this end, immobilisation of cyanobacteria and algae might provide a solution. The PFCE of such a photobiological fuel production process is likely to be much higher than that of a system where the biomass is harvested. Research on these “designer organisms” aims at 10 % PFCE, including a necessity for appropriate configuration of bioreactors. The future technologies, based on microbial electrosynthesis (MES) (see Rabaey and Rozendal 2010 for a review) as well as other hybrid systems (collectively called electrobiofuels), and on synthetic cell factories (living factories) and synthetic cell organelles, have a potential to reach even higher PFCEs.

### Second generation biofuels–sourced from biomass not competing with food production

These biofuels rely on using the biomass that is not suitable to be used as food. Scientific breakthroughs are expected in developing and exploiting both the new genome-based breeding systems and the biomass processing techniques towards production of second generation sustainable biofuels. It is important from societal and environmental viewpoints to keep investigating and improving the sustainability of these technologies. Second generation biofuels include (i) plants that are either specifically grown for bioenergy production (bioenergy crops) on marginal lands, i.e. areas not suitable for food production and on (ii) inedible parts of ordinary crops and forest trees that should be efficiently processed for bioenergy by improving the current technologies.

#### Bioenergy crops grown on marginal lands

There is a need to tailor plants and trees, using new genomic breeding techniques, for production of sugars, carbohydrates and oils that are more easily processed into biofuels than the natural ones, thus greatly contributing towards sustainable biofuel production. The combination of synthesis, transport, storage and use of molecules in plants during growth and development is collectively called “the plant energy system” and it determines the final yield and quality of plant products. Optimising the overall energy efficiency of plants/algae and focusing on production of the “most suitable target molecules in planta” for biofuel production will provide possibilities for remarkable breakthroughs in bioenergy/biofuel production. These opportunities for breakthroughs, however, entirely depend on the EU legislation, which should finally confirm that the products of New Breeding Techniques, when they do not contain foreign DNA, do not fall within the scope of GMO legislation (EASAC 2015). Acceptance of the new breeding techniques (EPSO 2015) is pivotal not only for food security but also for creating new plant varieties for bioenergy crops on marginal lands and for making the inedible parts of food-crop plants more suitable for biorefinery purposes and energy production. A message of an urgent need for more efficient plant breeding was clearly given to EU policy in a report launched by EASAC (2013) and in the recent statement (EASAC 2015). New breeding techniques include transgenesis (GM), cisgenesis, intragenesis, targeted mutagenesis, other transient introduction of recombinant DNA, as well as gene silencing and reverse breeding. Likewise, the epigenetic controls, gene variants and signals discovered will provide a new basis for sustainable productivity of bioenergy crops in marginal land areas and will produce plants to better tolerate changing climate conditions. Discovery of potential new breeding targets, on the other hand, is strongly connected to basic plant research at molecular level and on development of systems biology models to understand the function of plants.

There are already available quite well-defined traits that could be introduced into plants/algae to provide possibilities to enhance the energy efficiency and thus the biomass/biofuel production of plants and algae. Breeding targeted for production of suitable biofuel precursors in planta was already mentioned above. Other important traits include the following:

- Enhancement of photosynthesis. This concerns both the optimising of the light-capturing antenna size and the electron transduction efficiency to maximally sustain the CO_2_ fixation capacity (Maurino and Weber 2013). Big efforts are currently put to introduce the C4 carbon fixation pathway to less efficient C3 plants and similar benefits, i.e. elimination of energy-consuming photorespiratory pathways, can be obtained by introducing inorganic carbon pumps from photosynthetic microorganisms, like algae and cyanobacteria, to C3 plants (Singh et al. 2014). Introduction of entirely new CO_2_ fixation and metabolic pathways in plants is likewise possible by making use of approximately 5000 metabolic enzymes known to occur in nature (Bar-Even et al. 2010).
- Enhancement of stress tolerance of plants. It has been envisioned that, e.g. by intervention of ABA signalling by small molecules could help plants to combat stresses like drought, cold and soil salinity, typical to marginal lands suitable for bioenergy/biofuel crop cultivation. Similarly, many strategies are under development to improve plant strategies to combat against biotic stress. Further, the nutrient stress is often strongly limiting the yield and novel results from metabolome studies have recently given new hopes to design plants more tolerant, for example, against phosphorus deficiency.
- Breeding of plants for improvement of nutrient uptake and water use efficiency or the shaping of plant architecture to optimise light use efficiency are all important traits to improve biofuel production.

#### Biofuels from lignocellulosic biomass

Chemical and structural features of plant cell walls have hindered the progress in the production of cellulosic and hydrocarbon-based biofuels. Indeed, the development of commercial-scale cellulosic biofuel facilities has been slower than expected during the past decade(s).

Real scientific breakthrough would be a discovery of naturally occurring microbes that can break down lignin, a component of plants and trees, to give easier access to cellulose. Cellulose is a naturally occurring nanofibre structure within plant cell walls that keeps the cells together. To be used, it must be broken down to create sugar, which can then be converted into ethanol or other liquid fuels, like butanol and biodiesel. Currently, the biochemical processing of cellulosic biomass requires costly enzymes for sugar liberation. There is a need to unravel these tightly packed nanofibres more efficiently into soluble sugars using fewer enzymes. This could occur by developing advanced models to determine and improve enzyme kinetics behind the process. Likewise, providing tailored microbes with the capacity to ferment both cellulose and hemicellulose without the addition of expensive enzymes, the economics of cellulosic biofuels can be considerably improved.

Another approach to improve biofuel production from lignocellulosic biomass is to change the composition of the cell wall. Rapidly developing systemic research on chemistry, biochemistry and molecular biology of plant cell walls is paving the way towards this direction (Furtado et al. 2014). Genome-based new breeding technologies (discussed above) as well as the systemic and synthetic biology approaches will provide challenging opportunities for improvement of cell wall digestibility (Kalluri et al. 2014), and has a potential to significantly increase the PFCE from biomass.

Improved refinery technologies for biomass are likewise essential for decreasing the costs of second generation biofuels production. Developments towards making use of the entire biomass in the biorefinery process, thus closing the missing pieces in current biorefinery schemes, will greatly enhance the efficiency of biomass processing. Breakthroughs towards optimal refinery capacity are likely to be found either from novel pre-treatment processes of biomass or from improved biorefinery technologies. The integrated production of second generation biofuels from the pulp and other wood industries will likewise be an important outcome.

### Third generation biofuels–sourced from algal biomass

These biofuels include specific production of biodiesel and other algal-based biofuels like ethanol and biogas (Panbdey et al. 2014). Photosynthetic capture of CO_2_ in algal cultures for production of biomass and subsequent extraction of, e.g. biodiesel from algal cells has attracted lots of interest all over the world. The technology is still not economic and sustainable due to low PFCE of biodiesel production. This is partially due to the fact that enhanced biodiesel production generally occurs under stress conditions, which in turn reduces the growth and biomass production. Recent advances in metabolic engineering of algae to increase lipids without compromising growth are regarded as an important milestone towards sustainable biodiesel production (Trentacoste et al. 2013). Besides a number of various engineering approaches to increase algal fuel production (Ho et al. 2014), research also on algae biodiversity is likely to reveal interesting species with high capacity for fuel production (Maity et al. 2014). Combination of algal biofuel production with production of high-value chemicals, as well as the use of waste water and/or sea water as culture media and the development of more cost-effective bioreactors, are all technologies currently under heavy development and will in the near future make the algal biofuel production more profitable.

Moreover, the current biorefinery developments are mostly focused on use of carbohydrate containing plant material and waste (lignocellulose, bagasse, straw, industrial waste streams) in production of fuels, chemicals and materials, whereas the development of harvesting and processing systems for biofuel production from algal cultures has so far attracted less attention.

It is important to develop and genetically engineer cost-competitive algae fuels to overcome the cultivation, harvesting and processing problems (Medipally et al. 2015). Real breakthroughs still await solutions for breaking down technical barriers and accelerating the development of sustainable and affordable algae biofuels.

### Fourth generation biofuels–solar biofuels by synthetic biology technologies

Fourth generation biofuels take advantage of synthetic biology of algae and cyanobacteria (e.g. Berla et al. 2013; Hays and Ducat 2015; Scaife et al. 2015), which is a young but strongly evolving research field. Synthetic biology comprises the design and construction of new biological parts, devices and systems, and the re-design of existing, natural biological systems for useful purposes. It is becoming possible to design a photosynthetic/non-photosynthetic chassis, either natural or synthetic, to produce high-quality biofuels with high PFCE. For the first, second and third generation biofuels, the raw material is either biomass or waste, both being results of “yesterday’s photosynthesis” (yet not from fossil resources). While these biofuels often are very useful in a certain region or country, they are always limited by the availability of the corresponding organic raw material, i.e. the biomass, which limits their application on a global scale.

Fourth generation biofuels will be based on raw materials that are essentially inexhaustible, cheap and widely available. Photosynthetic water splitting (water oxidation) into its constituents by solar energy can become a large contributor to fuel production on a global scale, both by artificial photosynthesis (Inganäs and Sundström 2016) and by direct solar biofuel production technologies. Not only the production of hydrogen but also the production of reduced carbon-based biofuels is possible by concomitant enhanced fixation of atmospheric CO_2_ and innovative design of synthetic metabolic pathways for fuel production. The generation of “designer bacteria” with new useful properties requires revolutionary scientific breakthroughs in several areas of fundamental research.

The European Union bioeconomy strategy (European Commission 2012) highlights the importance of discovery research for the establishment of functional bioeconomy, and synthetic biology is foreseen as a key enabling technology for successful realisation of bioeconomy in replacement of fossil fuels. Synthetic biology will have the capacity to make bioeconomy much broader by providing means to produce numerous different biological compounds, including an array of various biofuels. There is also a worry about the decrease in EU’s biocapacity. If no actions will be taken, it is forecasted that consumption of bioresources will exceed their renewal capacity by 2030. Thus, it is considered extremely important to produce biofuels using minimal raw material resources in their production.

Fourth generation biofuels are produced (i) by designer photosynthetic microorganisms to produce photobiological solar fuels, (ii) by combining photovoltaics and microbial fuel production (electrobiofuels) or (iii) by synthetic cell factories or synthetic organelles specifically tailored for production of desired high-value chemicals (production currently based on fossil fuels) and biofuels.

#### Designer microorganisms in production of solar biofuels

**Fig. 1:**
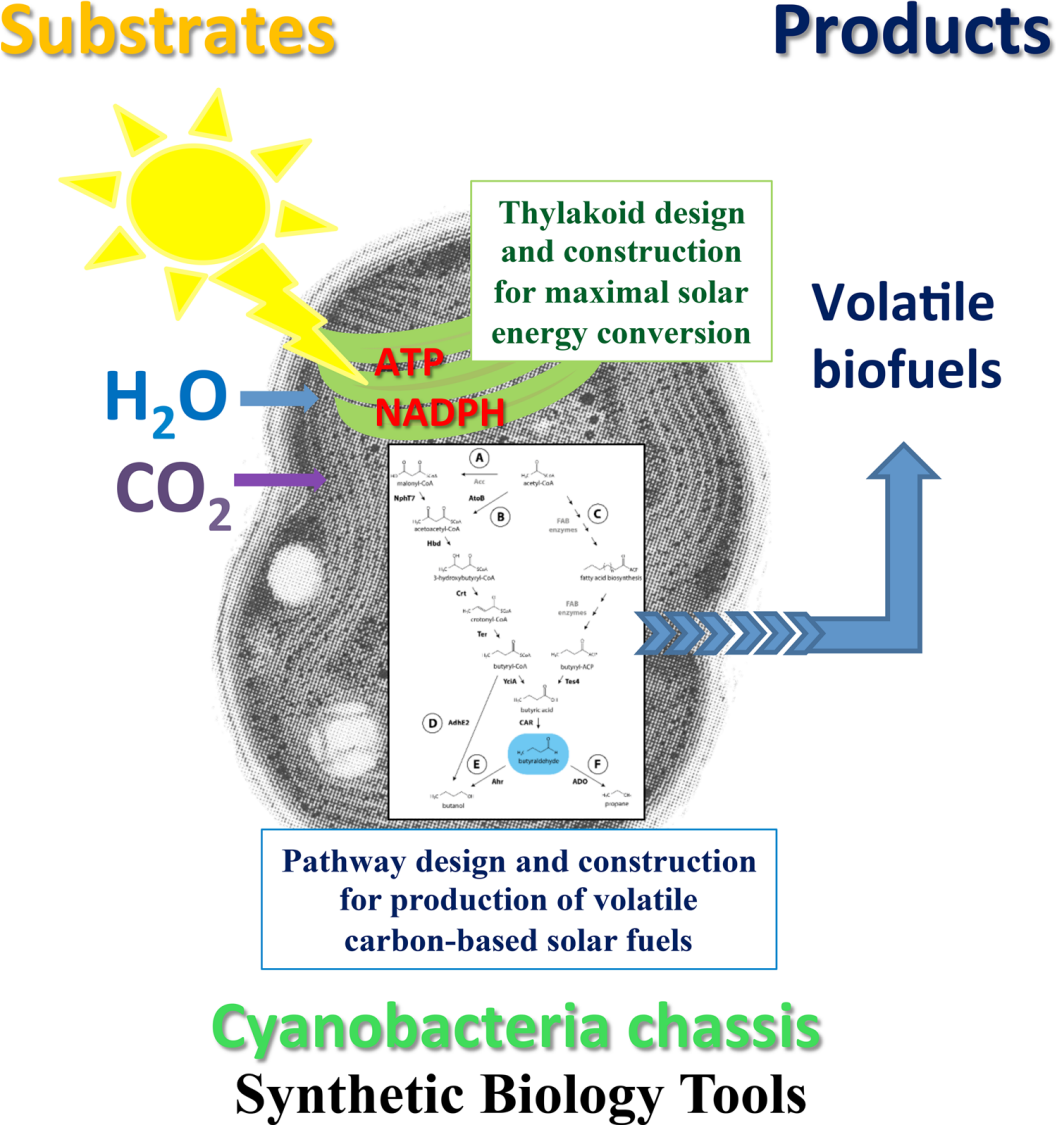
An example of development of a cyanobacteria chassis for production of carbon-based volatile direct solar biofuels. Living photosynthetic microorganisms are presently being tailored as designer cells, using synthetic biology tools, to efficiently convert solar energy to a fuel, using only CO_2_ and water as inexhaustible, cheap and widely available substrates

Key improvements in photon-to-fuel conversion efficiency (PFCE) as well as in the quality of the fuel will be based on generation of designer microorganisms. They produce a selected photobiological solar fuel, which is a non-fossil fuel made by direct solar energy conversion in photosynthetic microorganisms (algae or cyanobacteria), and are engineered, when necessary, to secrete the fuel (Fig. 1). Photobiological solar fuel is made in photosynthetic cell from solar energy using only water, or water and CO_2_ as raw materials, depending whether the produced fuel is hydrogen- or carbon-based fuel, respectively. Knowledge based on powerful biochemical and biophysical insights in natural photosynthetic light harvesting, water splitting, electron transfer, hydrogenases and carbon metabolism is critical for development of direct photobiological solar fuels. Microorganisms will be optimised for fuel production with synthetic biology approaches, metabolic engineering and organism design based on knowledge acquired by genomic research, molecular systems biology and extensive modelling research.

Most advanced research so far made towards direct photobiological solar fuel production is based on research with unicellular algae and cyanobacteria. Cyanobacteria are suitable as photosynthetic chassis for their well-developed genetic transformation technologies as well as for extensive knowledge on their light harvesting and electron transfer processes, on their metabolome and advanced modelling research of the entire cell. Cyanobacteria have been genetically engineered to produce various fuels and chemicals (e.g. H_2_, ethanol, isobutanol, isoprene, lactic acid). Introduction of various fermentative metabolism pathways to cyanobacteria cell by synthetic biology approaches has made it possible to produce biofuels directly from solar energy and Calvin–Benson cycle intermediates (Wijffels et al. 2013; Halfmann et al. 2014; Savakis and Hellingwerf 2015). Furthermore, the efficient secretion of product from the cells will increase the production capacity.

Breakthroughs in direct photobiological solar fuel production call for further research in design of light-harvesting systems, modelling and simulation of biological reactions and systems as well as in development of synthetic biology tools and production systems (Berla et al. 2013). Synthetic biology tools comprise the analysis, design and synthesis of biological systems based on well-defined and ideally orthogonal functional modules. Although the first results on direct photobiological solar fuels have proven their potential in fuel production, this field is still at the level of basic research. During the coming 10–20 years, it is expected that various photobiological solar fuels are gradually entering into the market.

#### Electrobiofuels

It is possible, through synthetic biology approaches, to establish new-to-nature hybrid production organisms that use renewable electricity and carbon sources for production of commodity chemicals and biofuels. A newly emerging field, MES (see Rabaey and Rozendal 2010 for a review) relies on the capability of certain microbes for direct electron capture from electrodes (e.g. from solar cells or any renewable electricity source) to assimilation of the reducing equivalents into metabolism, along with CO_2_ utilisation. Through MES, energy from solar cells can be turned into storable energy sources called electrofuels. Electrofuels allow renewables from all sources to be stored conveniently and efficiently as a liquid fuel. Bacteria, suitable for MES, transport electrons through the outer membrane via specific protein-embedded electron conduits. Direct electron transfer was estimated to be the most efficient mechanism for electron transfer from electrodes to microorganisms. Production of fuel butanol has already been demonstrated with the engineered MES organism *Clostridium ljungdahlii*. This technology may provide in the future an efficient way for storage of solar energy in the form of liquid fuel.

Recently, Torella et al. (Torella et al. 2015) demonstrated a novel and scalable integrative bioelectrochemical production system for isopropanol. The solar water-splitting catalyst, based on earth-abundant metals, was used to provide energy for growth of a bacterium *Ralstonia eutropha*. In metabolically engineered *Ralstonia*, the energy from water splitting could be diverted for production of isopropanol. Authors claim the highest bioelectrochemical fuel yield reported so far. Indeed, they provide an important proof-of-principle demonstration that inorganic and biological materials can be combined to achieve higher PFCE than either system can provide independently.

#### From current biorefineries to synthetic factories in production of solar biofuels

The design of synthetic factories and cell organelles for enhanced biofuel production is a future technology and currently just entering into intense developmental phase at the basic level of biofuel research. Likewise, the synthetic biology technology itself is still young and only a few truly synthetic examples have been realised by now (see Cameron et al. 2014 for a review). For optimal solutions, one needs to go beyond the known possibilities offered by biology by speeding up evolution and screening a massive number of artificial biological combinations. Thus, automation, microfabrication and measurement technologies are an integral part of a successful synthetic biology platform. Most importantly, engineering principles should be used to guide modelling, design and standardisation of the synthetic biological systems themselves and the entire development process so that optimisation can progress from the current trial-and-error situation to the design of truly programmable biofuel production systems.

Synthetic biology, after reaching maturity as a technology, will give tools and concepts that will make biology fully engineerable, and thereby make it possible to take full advantage of the diversity, functionality and specificity that biology can offer. Such research and technology development programmes should be established for construction of synthetic factories and organelles towards efficient biofuel production.

## Summary

Renewable biofuels, sourced from plants and photosynthetic microorganisms, are essential for a carbon–neutral bioeconomy. Sustainable biofuel production, however, still awaits many scientific breakthroughs. In the nearest future, (i) new technologies are needed for efficient biofuel production from non-edible parts of plants and algal biomass. This also includes breakthroughs (and political acceptance) in genomics-based breeding for increasing both the quantity and quality of biomass for biofuel production. Breakthroughs in (ii) direct solar biofuel research, aiming at biofuel production directly from photosynthetic microorganisms without a biomass phase and adapting synthetic biology technologies, are expected to significantly increase the photon-to-fuel conversion efficiency. (iii) Scientific breakthroughs in development of electrobiofuels, a combination of photovoltaics and microbial metabolic engineering, have a great potential in alleviation of energy storage problems. The most revolutionary future scenarios include (iv) the production of biofuels in “synthetic factories” together with high-value chemicals in order to make the production systems economically viable.
